# HIV-1 Subtype A Gag Variability and Epitope Evolution

**DOI:** 10.1371/journal.pone.0093415

**Published:** 2014-06-03

**Authors:** Syed Hani Abidi, Marcia L. Kalish, Farhat Abbas, Sarah Rowland-Jones, Syed Ali

**Affiliations:** 1 Department of Biological and Biomedical Sciences, Aga Khan University, Karachi, Pakistan; 2 Vanderbilt Institute of Global health, Vanderbilt University, Nashville, Tennessee, United States of America; 3 Department of Surgery, Aga Khan University, Karachi, Pakistan; 4 Nuffield Department of Medicine, University of Oxford, Oxford, United Kingdom; Chinese Academy of Sciences, Wuhan Institute of Virology, China

## Abstract

**Objective:**

The aim of this study was to examine the course of time-dependent evolution of HIV-1 subtype A on a global level, especially with respect to the dynamics of immunogenic HIV gag epitopes.

**Methods:**

We used a total of 1,893 HIV-1 subtype A gag sequences representing a timeline from 1985 through 2010, and 19 different countries in Africa, Europe and Asia. The phylogenetic relationship of subtype A gag and its epidemic dynamics was analysed through a Maximum Likelihood tree and Bayesian Skyline plot, genomic variability was measured in terms of G→A substitutions and Shannon entropy, and the time-dependent evolution of HIV subtype A gag epitopes was examined. Finally, to confirm observations on globally reported HIV subtype A sequences, we analysed the gag epitope data from our Kenyan, Pakistani, and Afghan cohorts, where both cohort-specific gene epitope variability and HLA restriction profiles of gag epitopes were examined.

**Results:**

The most recent common ancestor of the HIV subtype A epidemic was estimated to be 1956±1. A period of exponential growth began about 1980 and lasted for approximately 7 years, stabilized for 15 years, declined for 2–3 years, then stabilized again from about 2004. During the course of evolution, a gradual increase in genomic variability was observed that peaked in 2005–2010. We observed that the number of point mutations and novel epitopes in gag also peaked concurrently during 2005–2010.

**Conclusion:**

It appears that as the HIV subtype A epidemic spread globally, changing population immunogenetic pressures may have played a role in steering immune-evolution of this subtype in new directions. This trend is apparent in the genomic variability and epitope diversity of HIV-1 subtype A gag sequences.

## Introduction

Under selection pressures from host immunity, human immunodeficiency virus type 1 (HIV-1) rapidly mutates, which allows the amplification of escape mutations that enables the virus to evade the host's immune response [1]–[3]. Sequences of HIV-1 subtype A that were first deposited in the Los Alamos National Laboratory (LANL) HIV Sequence Database were of African origin, from mid-80s. In subsequent years, as subtype A viruses were disseminated globally, sequences from different parts of world were made available in the LANL database. In the past few decades, HIV-1 subtype A has established itself in certain regions of the world including Kenya, Uganda, Japan, Azerbaijan, Belgium, Botswana, Belarus, Congo, and recently, Afghanistan and Pakistan (http://www.hiv.lanl.gov/) [4], [5]. Analysis of the evolutionary patterns of a virus in the context of its host populations sheds light on the selection pressures on viruses that are associated with specific host immune milieus [6], [7]. This type of information is useful in designing vaccines and drugs against the virus.

In the current study, taking into account the sequences from years 1985 to 2010, we have analysed the divergence and evolution of HIV gag. We focus on both time-dependent as well as cohort-specific evolution of the HIV-1 subtype A gag gene, and present an analysis of evolutionary dynamics of HIV-1 subtype A on a global level, especially with respect to the evolution of gag epitopes.

## Methodology

### HIV-1 Subtype A Sequences

A total of 1,893 HIV-1 subtype A gag (spanning the p24 and p2p7p1p6 gene regions) DNA sequences from 19 countries (Rwanda, Kenya, Uganda, Sweden, Cyprus, Democratic Republic of Congo, Belarus, Tanzania, Russia, China, Ukraine, Italy, Australia, South Africa, Pakistan, Afghanistan, Spain, Zambia, Cameroon) were included in this study (Table S1). These sequences were downloaded from the HIV Los Alamos National Laboratory (LANL) Database (http://www.hiv.lanl.gov/). Out of these 1,893, a total of 94 sequences (19 Kenyan, 60 Pakistani and 15 Afghan) were from our previously studied cohorts [4], [8]–[10]. (Accession numbers: **Kenyan cohort sequences**: GU245698, GU245706, GU245710, GU245712, GU245713-15, GU245720, GU245726-27, GU245734, GU245736-37, GU245742-43, GU245748-50, GU245758. **Pakistani cohort sequences**: GU376767, GU376770, GU376772-74, GU376776-79, GU376781, GU376783-85, GU376788-91, JF804693, JF804697-21, JF804723-26, JF804730-37, JF804739-41, JF804743-44. **Afghan cohort sequences**: JF808139, JF808141, JF808144, JF808148, JF808151, JF808154, JF808156, JF808159, JF808163-65, JF808168-70, JF808172). The p24 and p2p7p1p6 region of gag was selected because the greatest number of sequences - both in terms of countries and years - were available for this region.

### HLA analysis of samples from Kenyan, Pakistani and Afghan cohort

Patient blood samples from our previously published cohorts in Kenya, Pakistan, and Afghanistan were collected after obtaining written informed consent. Ethical approval for this study was obtained from the Ethical Review Committee, Aga Khan University, Karachi, Pakistan. Using DNA samples from 94 Kenyan, Pakistani and Afghan patients, exons 2 and 3 of HLA class-I loci HLA-A, -B, and -C were amplified by PCR, based on the protocol described by *Bettinotti, et al.* [11], [12]. Approximately a 1 kb DNA fragment was amplified for each locus using exon-specific primers. Three different pairs of primers (HLA-A locus: 5′TTCTCCCCAGACGCCGAGGATGGCC3′, and 5′TGTTGGTCCCAATTGTCTCCCCTC3′; HLA-B locus: 5′ACCCACCCGGACTCAGAATCTCCT3′, 5′GGAGGCCATCCCCGGCGACCTAT3′, and 5′GGAGGCCATCCCCGGCGATCTAT3′; HLA-C locus: 5′GAGAAGCCAATCAGCGTCTCCGCA3′, and 5′GGAGATGGGGAAGGCTCCCCACT3′) were used for amplification of the region extending from 50-untranslated region (50-UTR) to the third exon of the HLA gene locus on human chromosome six [11], [12]. The PCR products obtained were commercially sequenced by Macrogen Inc., Korea, using primers for exons 2 (5′CACTCCATGAGGTATTTC3′) and 3 (5′GGCCAGGGTCTCACA3′). The same pairs of primers were used for all three loci, HLA-A, -B, -C. These sequences were used to assign HLA types employing the BLAST search option, available on the ImMunoGeneTics/Human Leukocyte Antigen(IMGT/HLA) database [13].

### Collection and year-wise grouping of HIV-I Subtype A sequences

From a total of 1,893 sequences, the oldest HIV-1 subtype A gag sequence was from 1985 (Uganda), while the most recent sequences were from 2010. These sequences were divided into five groups, all but one representing a 5-year block of sequences; the last block, 2005–2010, covered 6 years (inclusive of years 2005 and 2010). These groups were: 1985–1990, 1990–1995, 1995–2000, 2000–2005, and 2005–2010, comprising 133, 131, 1262, 198, and 169 sequences, respectively. All sequences were aligned using *MEGA5* software [14] and trimmed to a length of 447 bp. Unless stated otherwise, for all analyses performed, the same grouping of sequences was maintained.

### Phylogenetic Analysis

Evolutionary relationships among the HIV-1 subtype A sequences were analysed using a Maximum likelihood (ML) tree, constructed with 1,893 subtype A gag sequences using *MEGA5* [14]. For the ML tree, the following parameters were used: substitution model – GTR; rates among sites – Gamma distributed with Invariant sites (G+I); number of discrete gamma categories – 5 (default value); ML heuristic method – Nearest-Neighbor-Interchange (NNI); initial tree for ML – NJ/BioNJ; test of phylogeny – bootstap (100 replicates). For this analysis, the SIV_cpz_ gag sequence from 1988 (Accession number: X52154) was used as outgroup to root the tree. The ML tree was refined using *FigTree* v1.3.1 (http://tree.bio.ed.ac.uk/software/figtree/) software. The software artificially compresses the tree to fit in large number of sequences.

### Effective population size and time of the most recent common ancestor (tMRCA)

A Bayesian Markov Chain Monte Carlo (MCMC) inference was applied to estimate the effective population size and tMRCA of HIV subtype A, using *BEAST* v1.7.4 software [15]. For this analysis, a total of 113 sequences from different countries from 1985 to 2010 were used. Sequences were selected using the approach described by *Novitsky, et al.* [6], where a limit was set to 5–6 sequences per country and year-groups. If 5 or less sequences were represented in a country all of them were included in the analysis. Bayesian analysis was performed using the following parameters: uncorrelated lognormal relaxed molecular clock model [16], general time-reversible (GTR) nucleotide substitution model, estimated base frequencies, and gamma distribution model for heterogeneity among nucleotide sites. The analysis was performed using demographic models of constant population size and Bayesian skyline plot [17]. The MCMC chain length was set at 2×10^8^, which gave an effective sample size (ESS) of >200. MCMC sample analysis and Bayesian skyline plot construction were performed using *Tracer* v1.7.4. In a separate experiment, in order to control the effect of high G→A substitutions on effective population size and tMRCA, all sequences with high G→A substitutions (mostly Afghan and Pakistani sequences) were removed and the Bayesian analysis was performed again.

### Time-dependent genomic variability

In order to evaluate time-dependent changes in HIV subtype A genomic variability, G→A substitutions and Shannon entropy analyses were performed. The Shannon entropy of subtype A gag gene sequences from the five different year-groups was calculated with as well as without the sequences from our three cohorts, using an online tool available at the Los Alamos National Laboratory (LANL) HIV Sequence Database: http://www.hiv.lanl.gov/content/ sequence/ENTROPY/entropy_one.html.

G→A substitution analysis of the HIV sequences was performed using Hypermut (both 2.0 and old versions) tool available at the LANL HIV Sequence Database (http://www.hiv.lanl.gov/content/sequence/HYPERMUT/hypermut.html) [18]. For this analysis, HIV subtype A reference sequences from years 1985, 1992, and 2003 were retrieved from LANL HIV Sequence Database and were used as follows: 1985 sequence was used for the year group 1985–1990, 1992 sequence for year-groups 1990–1995 and 1995–2000, and 2003 sequence for 2000–2005 and 2005–2010 [19]. Since a reference sequence for year 2005 through 2010 was not available in the LANL database, the 2003 reference sequence was used for 2000–2005 as well as for 2005–2010 block. The values for each step (G→A substitutions and Shannon entropy) were entered into the *GraphPad* software to determine the mean for each group, while statistical significance between year-wise groups was calculated using one-way ANOVA with Tukey's multiple comparison test, using *GraphPad* software.

### Prediction of CTL epitopes and HLA restriction

Consensus gag *gene* sequences for each year-group were translated into amino acid sequences using *Expasy* translate tool. These protein sequences were then used to predict CD8+ T cell epitopes using *CTLPred* software [20]. A similar strategy was used to predict CD8+ T cell epitopes in sequences from Kenyan, Pakistani and Afghan cohorts. Additionally, for sequences from our study cohorts, the MHC restriction for each epitope was predicted using *ProPred-I* [21] and *nHLAPred* software [22].

Since HLA data was available for our three study cohorts, population and allele frequencies of HLA for these cohorts were calculated using online HLA frequency tool available at Los Alamos website (Los Alamos National Laboratory, http://www.hiv.lanl.gov). This tool computes the 2-sided exact Fisher's p-value for each HLA in a given population. False discovery was corrected by Storey's q-value. HLA alleles were represented in the Kenyan, Afghan and Pakistani cohorts with a frequency of 10% or above were marked as ‘predominant HLA alleles’ in the three cohorts.

Sequences from our three study cohorts were part of 2005–2010 year-group, when analysis for genetic variability (i.e., G→A substitutions and Shannon entropy) and epitope prediction was carried out. To confirm whether observations for genetic variability and immunodynamics analysis were also reflected in a population-specific manner, sequences from these three cohorts were also analysed separately.

## Results

### Analysis of the phylogenetic relationships, origin, and global epidemic dynamics of HIV-1 subtype A

**Figure 1:**
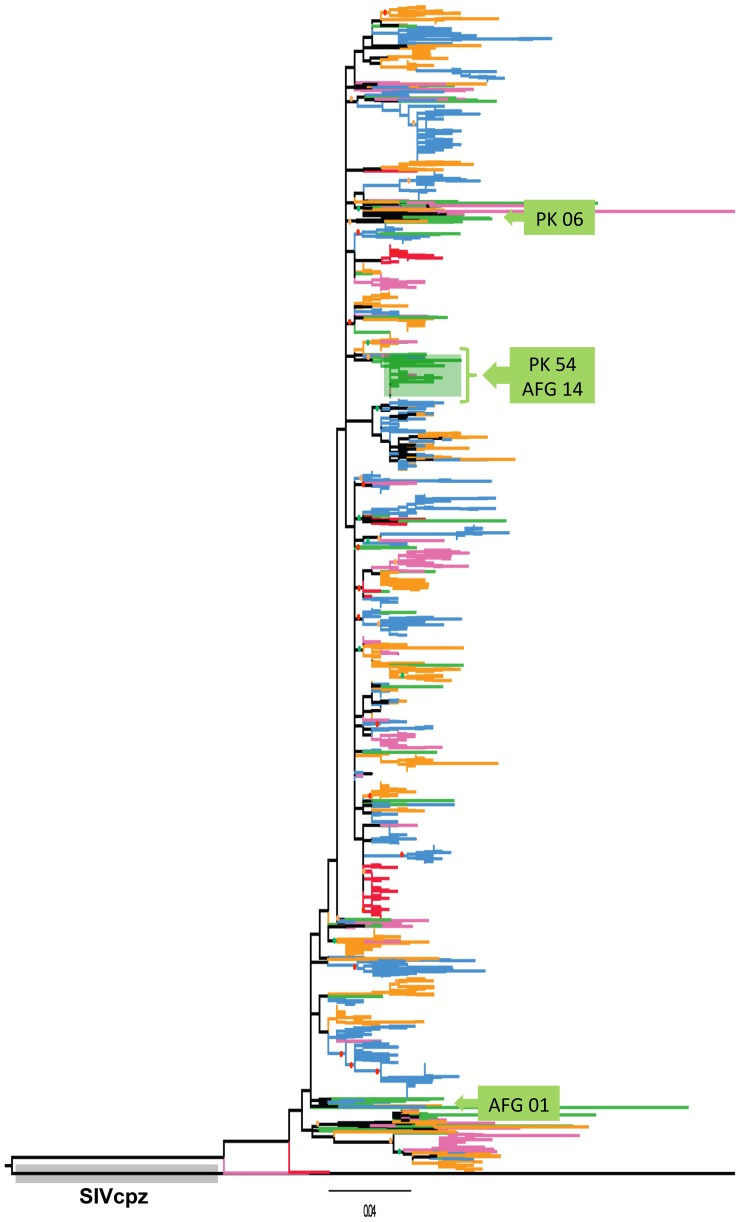
Maximum likelihood (ML) tree of HIV-1 subtype A gag gene sequences. ML tree was used to infer phylogenetic relationship among the 1,893 HIV-1 subtype A gag sequences submitted to the LANL HIV Sequence Database, representing the years 1985 to 2010. Grey shaded area shows the outgroup sequence SIV*cpz* gag, that was also used to root the tree. Sequences from year-groups 1985–1990, 1990–1995, 1995–2000, 2000–2005, and 2005–2010 are shown in red, blue, orange, pink and green colour, respectively. The Green shaded area indicates sequences from Afghanistan and Pakistan that were found to group together. The green boxes indicate position and number of Afghan (AFG) and Pakistani (PK) sequences present in the tree. Nodes with bootstrap values >90, >80 and >70 are indicated by red, green and orange circles, respectively.

Phylogenetic relationships among the HIV subtype A gag sequences were inferred through a Maximum Likelihood (ML) tree. For this analysis, an SIV_cpz_ gag sequence was used as an outgroup to root the ML tree. Analysis of the tree structure revealed that HIV-1 subtype A gag sequences from the different year-groups were scattered throughout the phylogenetic tree, without any apparent relationship between branching topology and time of sampling (Figure 1). There are significant clusters in the tree supported by bootstrap values greater than 70%. However, since 90% of the sequences in the tree are from Kenya, these clusters likely represent some Kenya transmission networks. However, there was one significant cluster in the tree that was comprised of sequences from just Pakistan and Afghanistan (Figure 1, green shaded square).

**Figure 2:**
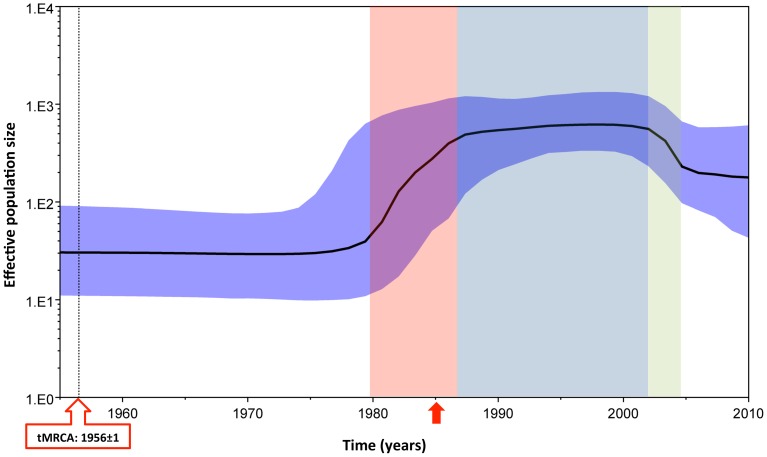
HIV-1 subtype A effective population size and time for most recent common ancestor. Bayesian Skyline plot, based on a ‘relaxed clock’ coalescent framework analysis, was constructed using 113 sequences (representing all years and countries). X-axis represents time in years, while Y-axis shows the effective population size. The thick black line represents the median, while the blue band represents 95% highest posterior density (HPD) intervals. The tMRCA of HIV-1 subtype A is indicated by a black dotted line and red box, while the time for the 1985 sequence, which was the oldest reported HIV subtype A strain in the LANL Sequence Database) is indicated by a red arrow. Red, light blue and green shaded areas represent the period of increase in viral effective population size, plateau phase, and decline, respectively.

To explore the relationship between gag divergence and epidemic dynamics, Bayesian Skyline analysis was performed. Using this analysis, the most recent common ancestor (tMRCA) of HIV-1 subtype A was estimated to be around 1956±1 (Figure 2, black dotted line). Compared to the origin of tMRCA, the Bayesian Skyline plot identified at least an initial 10-fold growth in viral effective population size (correlating with effective number of infections and/or transmission opportunities [23]) during the early to mid-1980s (Figure 2, red area). Effective population size became stationary from the mid-to-late 1980s until the early 2000s (Figure 2, blue area), followed by a period of decline, where an approximate 3-fold decrease was observed (Figure 2, green area). The effective population size became stationary from about 2004 onwards. Sequences with high G→A substitutions can introduce bias in the Skyline analysis by introducing noise in the overall data. To control for the effect of high G→A substitutions on the overall effective population size and tMRCA, in a separate analysis all sequences with high G→A substitutions were removed and Bayesian analysis was performed again. The resulting Skyline plots, and tMRCA were found to be similar to the one where sequences with high G→A substitutions were retained in the analysis (data not shown).

### Time-dependent genetic evolution of HIV subtype A gag

**Figure 3:**
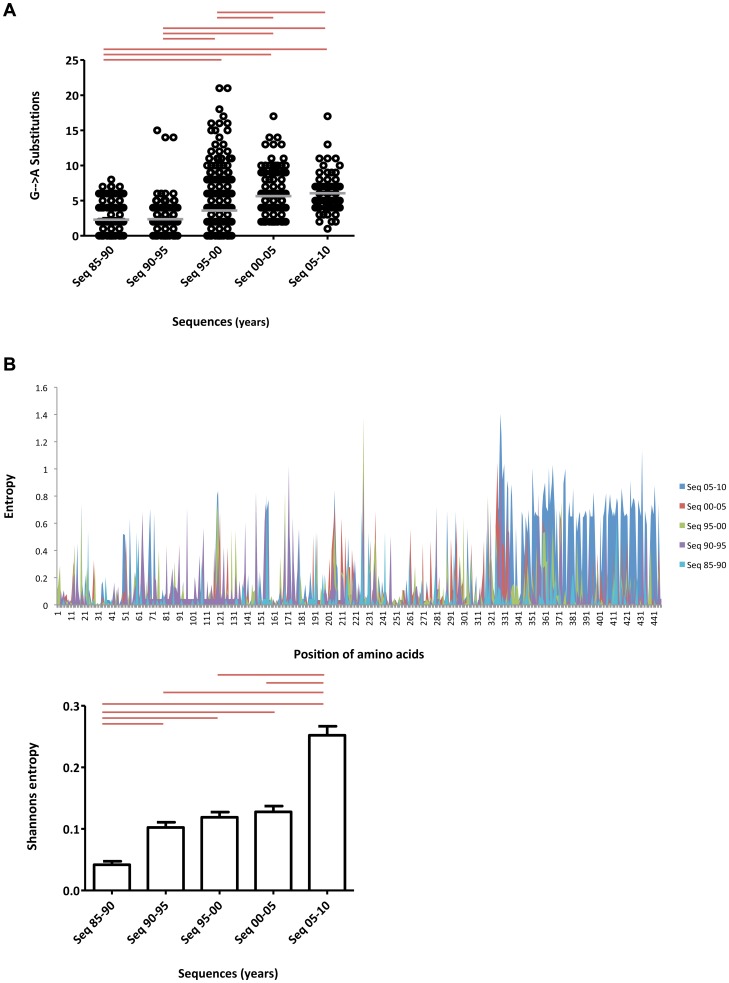
Time-dependent changes in HIV-1 subtype A genomic variability. HIV-1 gag variability was measured for all five year-groups using a) G→A substitutions and b) Shannon entropy. **a**) G→A substitutions: Small black circles represent number of substitutions for each sequence; grey lines show the mean for a particular year-group. The red line over the scatter plot represents statistically significant (p<0.001) differences between groups. **b**) Shannon entropy analysis: The vertical axis represents entropy scores, while the horizontal axis shows position of amino acids in the gag gene. Shannon entropy scores for each year-group are represented in the following colors: 1985–90 (turquoise), 1990–95 (purple), 1995–00 (green), 2000–05 (red) and 2005–2010 (blue) **Insert**: Mean entropy score for each year-group was calculated and plotted using GraphPad software. Red line over bars represents a statistically significant difference between the groups (p<0.001). Error bars represent the standard error of the mean.

In order to analyse HIV-1 subtype A gag variability over the study period, we performed G→A substitutions and Shannon entropy analysis on subtype A gag sequences grouped into five 5-year sets. It was observed that during the first ten years of the epidemic (1985–1995), G→A substitutions were at a low level with a mean of about two substitutions per sequence (Figure 3a)., A significant (p<0.001) increase in G→A substitutions was observed from 1995 though 2000 compared to the earlier time-periods, which continued into the last five years (2005–2010) of epidemic (Figure 3a). Shannon entropy analysis revealed a significant (p<0.001) increase in entropy from years 1985–90 to 1990–95 (Figure 3b, Figure 3b insert), followed by a plateau phase until 2005, when the change in entropy was non-significant. Compared to all other year-groups, the last six years (2005–10) were marked by a sharp, significant (p<0.001) increase in entropy (Figure 3b, Figure 3b inset). It was interesting to note that this increase in entropy was most profound in the p2p7p1p6 region of HIV-1 gag (Figure 3b, blue peaks).

### Time-dependent global immunodynamics of HIV-1 subtype A gag

To analyse the diversity of gag epitopes over time, the number of CTL epitopes, their HLA restriction and epitope variability (total number of mutations in all epitope sequences in a given year-group/total number of epitopes in a year-group) were evaluated over the years 1985–2010.

**Figure 4:**
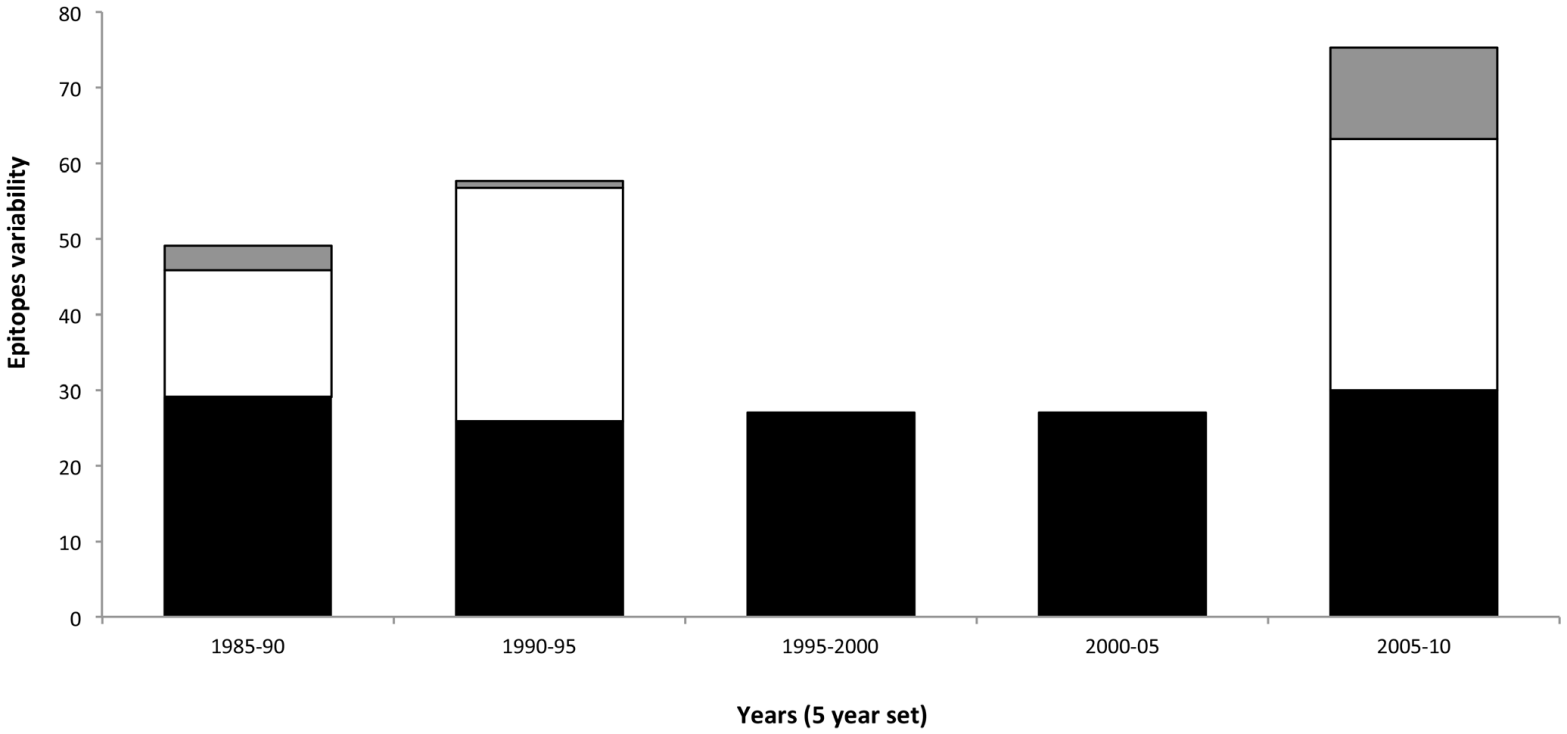
Divergence and evolution of HIV-1 subtype A gag epitopes. Bar chart summarizing epitope data for each year-group. Black bars show the total number of gag epitopes observed for each year-group, white bars represent epitope variability (total number of mutations in all epitope sequence in year-group/total number of epitopes in a year-group), and grey bars indicate novel epitopes that were observed for each year-group.

Consistent with G→A substitution and Shannon entropy analyses, a time-dependent increase in the diversity of gag epitopes was observed. Only three unique epitopes were observed during 1985–1989 (Figure 4 and Figure S1, red highlighted epitopes), while only one was observed in 1990–94 (Figure 4 and Figure S1, blue highlighted epitopes). This was followed by 10 years (1995–2004) where no novel epitopes and only negligible epitope variability was observed. During the last six years of the study period (2005–2010), the highest number (12) of unique epitopes was observed (Figure 4 and Figure S1, green highlighted epitopes). Similarly, in the same year-group, the highest increase in epitope variability (33%) was observed, compared to the rest of the study periods.

### Population-specific immunodynamics of HIV-1 subtype A gag

To confirm whether genetic variability observations in the preceding analysis were also reflected in a population-specific manner, we analysed HIV-1 subtype A gag variability and gag epitope data from the Kenyan, Pakistani, and Afghan cohorts that we have been studying for the past five years [4], [8], [9], [24], [25]. Sequences from these three cohorts are predominantly found in 2005–2010 year-group.

**Figure 5:**
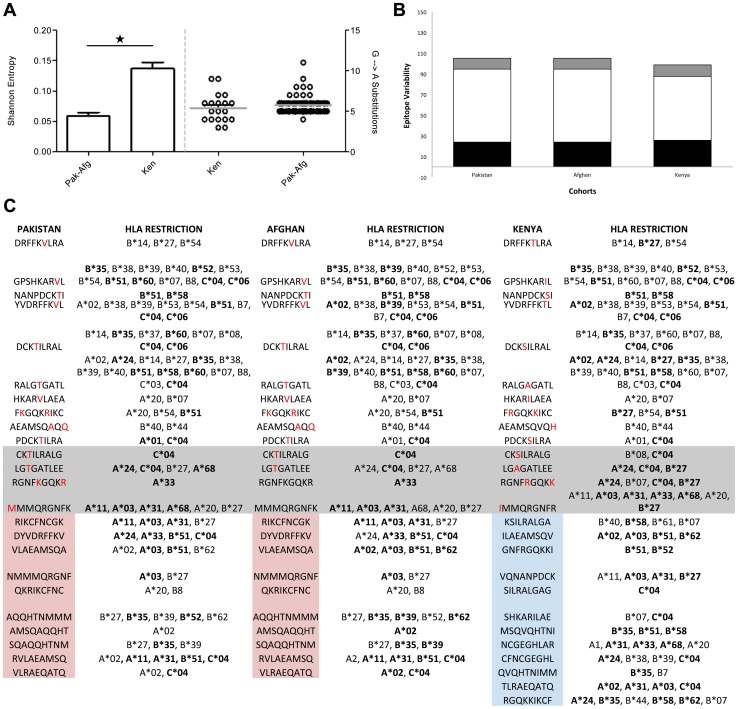
Cohort-specific analysis of HIV-1 subtype A gag gene and epitope variability. **A**) Genomic variability was measured for sequences from our Afghan, Pakistani and Kenyan cohorts using **A**) G→A substitutions and Shannon entropy. G→A substitutions (right): Small black circles represent the number of substitutions for each sequence, while the grey lines show the mean for a particular cohort. Shannon entropy analysis (left): A single star over bars represents a statistically significant (p<0.001) difference between the groups. The red bars represent standard error of mean. **B**) Bar chart summarizing the epitope data for Kenyan, Pakistani and Afghan study cohorts. Black bars show the total number of gag epitopes observed for each cohort, white bars represent epitope variability (total number of mutations in all epitope sequence in cohort/total number of epitopes in a cohorts), and the grey bars show the unique epitopes observed for each year-group, **C**) All epitopes and their corresponding predicted HLA restriction patterns that were identified in HIV-1 subtype A gag sequences for the three study cohorts. Epitopes observed in Pakistan and Afghan cohorts are highlighted red, while epitopes specific to Kenyan cohort are highlighted blue. Red letters denote mutations observed in a particular epitope, while the grey highlighted rows represent epitopes for which loss or gain of HLA restriction pattern was observed. HLA highlighted in bold indicate alleles that were predominantly observed in the study cohorts (*see Methods*).

It was observed that these Kenyan sequences had significantly higher Shannon entropy (p<0.001) as compared to Pakistani and Afghan sequences (Figure 5a, bar graphs). G→A substitutions analysis revealed that Pakistani and Afghan sequences had a slightly higher number of G→A substitutions as compared to Kenyan sequences, however, the difference was not significant (Figure 5a, scatter plot).

To analyse the diversity of gag epitopes in the three cohorts, the number of CTL epitopes, their HLA restriction profile, and epitope variability (total number of mutations in all epitope sequences in a given cohort/total number of epitopes in a cohort) was evaluated. In Pakistani, Afghan, and Kenyan cohorts, respectively, 10, 10, and 11 unique gag epitopes were observed (Figure 5c). The repertoire of epitopes was identical between Pakistani and Afghan cohort, while distinct from those in the Kenyan cohort (Figure 5c). Since a major portion of the HIV sequences from the Pakistani and Afghan cohorts are phylogenetically closely linked (Figure 1), observing a high degree of similarity in their epitope pattern was not surprising. Furthermore, since sequences in the 2005–2010-year group were predominantly from these three cohorts, the epitope profile of this year-group considerably overlapped with that of the three cohorts (compare Figure 5c and Figure S1).

The HLA restriction pattern predicted for the gag epitopes from Afghan, Kenyan and Pakistani cohorts revealed cohort-specific differences in HLA restriction profiles, which were identical in Afghan and Pakistani cohorts, but distinct in Kenyan cohort (Figure 5c; grey highlighted rows).

## Discussion

In this study, using all 1,893 sequences from the HIV-1 subtype A gag p24 and p2p7p1p6 region submitted to the LANL HIV Sequence Database, representing 19 countries and years 1985 through 2010, we examined the time-dependent evolution of HIV-1 subtype A. Out of these sequences, 94 were from our Afghan, Kenyan and Pakistani cohorts, and we used these sequences to perform population-specific immunodynamics of HIV-1 subtype A gag gene region. Our analysis was specifically focused on the genetic variability and epitope diversity in this gene region.

Phylogenetic analysis revealed that HIV-1 subtype A gag sequences from different year-groups were dispersed throughout the tree suggesting on-going evolution or diversification of the virus throughout the epidemic [6]. While we identified dispersed, significant clusters of sequences throughout the tree, most of the sequences (about 90%) in our analysis were from Kenya – which most likely skewed the overall phylogeny to reflect Kenyan transmission clusters. We also found that the majority of sequences from Afghanistan and Pakistan (year group 2005–10), grouped together in a single monophyletic cluster (Figure 1, green highlight). Clustering of Afghan and Pakistani sequences reflects the close relationship between the subtype A epidemic in the two countries, as evident from our previously published data [4], [26]. The HIV strains in Afghan refugees and Pakistani HIV-infected communities share phylogenetic relationship most likely because the routes of transmission of infection are the same between Pakistani patients and the Afghan refugees residing in Pakistan [4], [26].

Bayesian analysis indicated that the most recent common ancestor of what we call HIV-1 subtype A viruses arose about 1956. Based on effective population size, from 1956 until about 1980, there was a low and stable level (effective population size: 1×10^1.5^) of HIV transmissions. Beginning in 1980, there was a rapid, epidemic-level expansion of HIV-infections which peaked about 1987 [23]. Following by a stationary phase or stabilization of the epidemic that lasted until early 2000, followed by a short period of decline until about 2004, after which the epidemic began to plateau once again.

HIV gag genetic variability (measured using G→A substitutions and entropy) during the first 10 years (1985–95) of the epidemic increased significantly (p<0.001) from the mid-1990s onwards, eventually reaching a peak between 2000–2010. This trend was also observed for subtype A gag epitope patterns where novel epitopes and epitope mutations reached a peak between 2005–2010.

Following HIV-1 infection, the host immune system strives to control the viral load, while the virus struggles to escape the host defence by incorporating immune escape mutations into its genome, driven by selection pressures, resulting in increased variability in the viral genome [27]. The parameters for measuring genetic variability used in this study were Shannon entropy and G→A substitutions. Shannon entropy is a measure of the probability of acquiring mutations, including epitope-related mutations, in a given set of genomic sequences [27]. On the other hand, G→A substitutions, introduced into the viral genome by the host's secondary viral control factors, such as APOBEC protein [27], also contribute to the genetic variability of the virus [28].

Evaluation of epidemic dynamics and genetic variability helps in determining the course of viral evolution. The decrease observed in viral effective population size after 2004, combined with an increase in gag gene and epitope variability from 2005 onwards might be reflective of subtype A strains in populations with immunogenetic backgrounds that may increase the amount of immunological escape mutations while negatively affecting viral replication fitness [29]. For instance, it may be an indication of the introduction of subtype A viruses in populations having a predominance of protective alleles, such as HLA B*51 [30], as observed in the Afghan and Pakistani populations we studied [4], [5], [24], [25], where HLA data was available. The observed variability in the gag gene, as well as in the epitope pattern, could be driven, in part, by the varying HLA backdrops the viruses encountered as the HIV epidemic travelled through different populations. In addition, continuing HIV evolution over time, as well as founder effects arising from the introduction of new, unique subtype A strains within a population, also contribute to diversity in gag. This was especially apparent in our three cohort analysis (Figures 5a and 5b). For example, epitope LGAGATLEE, observed in Kenyan cohort, was predicted to be restricted by HLA A*24/C*04/B*27. This epitope was mutated to LGTGATLEE in Pakistani and Afghan cohorts, changing the predicted restriction to HLA A*68 (Figure 5c, grey highlighted rows).

A relatively high value of sequence entropy and epitope variation observed in Pakistani, Afghan and Kenyan sequences in the years 2005–2010 may indicate a transient phase in the virus' genetic evolution, which may later be followed by a refinement and amplification of discreet escape mutants, which is probably indicated by the stabilization in viral effective population size observed from about 2004 onwards. Keeping in mind the limited number of sequences available from 2004-onwards, it is likely that this analysis represents an incomplete picture of the status of epidemic from 2004 onwards. As more subtype A sequences from this period become available, similar analyses will give a better insight into how the genetic and immunological pressures will shape the HIV epidemics in the upcoming years.

A year-wise analysis of epitope sequences from the same populations will help in deciphering the true nature of this phenomenon. Further analyses will provide a better understanding of the directions in which the HIV-1 epidemics continue to evolve. This information will be crucial in anticipating prevention and control strategies for subtype A-infected patients, especially in populations where the epidemic is newly emerging.

## Supporting Information

Table S1 **Sequences used in this study.** A total of 1893 sequences were used in the study. The table represents, for each year-group: total number of sequences, countries from where the sequences were deposited and number of sequences from each country. Sequences from our Kenyan, Pakistani and Afghan cohorts are shaded grey.
(DOCX)

Figure S1 **Time-dependent evolution of HIV-1 subtype A gag epitopes.** All epitopes were identified in the HIV-1 subtype A gag sequences for each year-group (columns 1–5), and epitopes found to be unique in each year-group are highlighted: 1985–90 (red), 1990–95 (blue) and 2005–2010 (green). Red letters denote mutations observed in a particular epitope when compared with similar epitopes in other year-groups.
(DOCX)
