# Supplementary material for: HIV-1 Subtype A Gag Variability and Epitope Evolution
Source: PLoS One. 2014 Jun 3;9(6):e93415. doi: 10.1371/journal.pone.0093415 (PMC4043486; doi:10.1371/journal.pone.0093415)
Supplement: Table S1 — Sequences used in this study. A total of 1893 sequences were used in the study. The table represents, for each year-group: total number of sequences, countries from where the sequences were deposited and number of sequences from each country. Sequences from our Kenyan, Pakistani and Afghan cohorts are shaded grey. (DOCX) [file pone.0093415.s001.docx]

**Supplemantry Table 1**

| **Year- group** | **Total sequences** | **Countries represented in each year-group** | **Number of sequences** |
| --- | --- | --- | --- |
| 1985-90 | 133 | Uganda | 1 |
|  |  | Kenya | 132 |
| 1990-95 | 131 | Kenya | 114 |
|  |  | Rwanda | 6 |
|  |  | Uganda | 5 |
|  |  | Sweden | 1 |
|  |  | Cyprus | 5 |
| 1995-00 | 1262 | Kenya | 1256 |
|  |  | Democratic Republic of the Congo | 3 |
|  |  | Belarus | 1 |
|  |  | Tanzania | 1 |
|  |  | Sweden | 1 |
| 2000-05 | 198 | Kenya | 170 |
|  |  | Russia | 4 |
|  |  | China | 1 |
|  |  | Ukraine | 1 |
|  |  | Democratic Republic of the Congo | 1 |
|  |  | Italy | 1 |
|  |  | Australia | 2 |
|  |  | Tanzania | 15 |
|  |  | Cyprus | 2 |
|  |  | South Africa | 1 |
| 2005-10 | 169 | Kenya | 14 |
|  |  | Kenya | 19 |
|  |  | Pakistan | 60 |
|  |  | Afghanistan | 15 |
|  |  | Cyprus | 38 |
|  |  | Russia | 10 |
|  |  | Tanzania | 1 |
|  |  | Spain | 3 |
|  |  | Uganda | 4 |
|  |  | Zambia | 1 |
|  |  | Cameroon | 2 |
|  |  | Rwanda | 2 |
