# Supplementary material for: HIV-1 Subtype A Gag Variability and Epitope Evolution
Source: PLoS One. 2014 Jun 3;9(6):e93415. doi: 10.1371/journal.pone.0093415 (PMC4043486; doi:10.1371/journal.pone.0093415)
Supplement: Figure S1 — Time-dependent evolution of HIV-1 subtype A gag epitopes. All epitopes were identified in the HIV-1 subtype A gag sequences for each year-group (columns 1–5), and epitopes found to be unique in each year-group are highlighted: 1985–90 (red), 1990–95 (blue) and 2005–2010 (green). Red letters denote mutations observed in a particular epitope when compared with similar epitopes in other year-groups. (DOCX) [file pone.0093415.s002.docx]

1985-90	1990-95	1995-2000	2000-05	2005-10
DRFFKTLRA	DRFFKTLRA	DRFFKTLRA	DRFFKTLRA	DRFFKALRA
YVDRFFKTL	YVDRFFKTL	YVDRFFKTL	YVDRFFKTL	YVDRFFKAL
NANPDCKSI	NANPDCKSI	NANPDCKSI	NANPDCKSI	NANPDCKTI
ATQEVKGWM	ATQDVKNWM	ATQEVKGWM	ATQEVKGWM	ATQEVKGWM
QATQEVKGW	QATQDVKNW	QATQEVKGW	QATQEVKGW	QATQEVKGW
DCKSILRAL	DCKSILRAL	DCKSILRAL	DCKSILRAL	DCKTILRAL
VLAEAMSQV	VLAEAMSQV	VLAEAMSQV	VLAEAMSQV	VLAEAMSQA
IMMQRGNFK	IMMQRGNFR	IMMQRGNFR	IMMQRGNFR	MMMQRGNFR
RAEQATQEV	RAEQATQDV	RAEQATQEV	RAEQATQEV	RAEQATQEV
AEAMSQVQH	AEAMSQVQH	AEAMSQVQH	AEAMSQVQH	AEAMSQAQQ
RALGAGATL	RALGPGATL	RALGPGATL	RALGPGATL	RALGTGATL
GWMTETLLV	NWMTETLLV	GWMTETLLV	GWMTETLLV	GWMTETLLV
RGNFKGQKR	RGNFRGQKR	RGNFRGQKR	RGNFRGQKR	RGNFRGQKR
	EQATQDVKN	EQATQEVKG	EQATQEVKG	EQATQEVKG
PDCKSILRA	PDCKSILRA	PDCKSILRA	PDCKSILRA	PDCKTILRA
LGAGATLEE	LGPGATLEE	LGPGATLEE	LGPGATLEE	LGTGATLEE
CKSILRALG	CKSILRALG	CKSILRALG	CKSILRALG	CKTILRALG
.	RGQKRIKCF	RGQKRIKCF	RGQKRIKCF	.
AEQATQEVK	.	AEQATQEVK	AEQATQEVK	AEQATQEVK
GNFKGQKRI	GNFRGQKRI	GNFRGQKRI	GNFRGQKRI	.
EGHLARNCR	EGHLARNCR	EGHLARNCR	EGHLARNCR	.
VQNANPDCK	VQNANPDCK	VQNANPDCK	VQNANPDCK	.
QVQHTNIMM	QVQHTNIMM	QVQHTNIMM	QVQHTNIMM	.
MSQVQHTNI	MSQVQHTNI	MSQVQHTNI	MSQVQHTNI	.
KEGHLARNC	KEGHLARNC	KEGHLARNC	KEGHLARNC	.
KCFNCGKEG	KCFNCGKEG	KCFNCGKEG	KCFNCGKEG	.
TLRAEQATQ	.	TLRAEQATQ	TLRAEQATQ	.
SILRALGAG	.	.	.	.
KSILRALGA	.	.	.	.
KGQKRIKCF	.	.	.	.
.	KNWMTETLL	.	.	.
.	.	.	.	DYVDRFFKA
.	.	.	.	ALRAEQATQ
.	.	.	.	RVLAEAMSQ
.	.	.	.	GKEGHLARN
.	.	.	.	NMMMQRGNF
.	.	.	.	CGKEGHLAR
.	.	.	.	QKRIKCFNC
.	.	.	.	AQQHTNMMM
.	.	.	.	FRGQKRIKC
.	.	.	.	AMSQAQQHT
.	.	.	.	SQAQQHTNM
.	.	.	.	MQRGNFRGQ
